# Access to Norwegian healthcare system – challenges for sub-Saharan African immigrants

**DOI:** 10.1186/s12939-019-1027-x

**Published:** 2019-08-14

**Authors:** Vivian N. Mbanya, Laura Terragni, Abdi A. Gele, Esperanza Diaz, Bernadette N. Kumar

**Affiliations:** 1 Department of Community Medicine and Global Health, Institute of Health and Society, Faculty of Medicine, University of Oslo, Oslo, Norway; 2 Department of Nursing and Health Promotion, Faculty of Health Sciences, Oslo Metropolitan University, Oslo, Norway; 3 Unit for Migration Health, Norwegian Institute of Public Health Oslo, Oslo, Norway; 4 Department of Global Public Health and Primary Care, University of Bergen, Bergen, Norway

**Keywords:** Sub-Saharan Africa, Immigrants, Norway, Access to healthcare services, Challenges

## Abstract

**Background:**

Immigrants face barriers in accessing healthcare services in high-income countries. Inequalities in health and access to healthcare services among immigrants have been previously investigated. However, little is known on the sub-Saharan African immigrants’ (SSA) access to the Norwegian healthcare system.

**Methods:**

The study had a qualitative research design. We used the snowball technique to recruit participants from networks including faith-based organizations and cultural groups. Forty-seven qualitative in-depth interview and two focus group discussions with immigrants from sub-Saharan African were conducted from October 2017 to July 2018 in Oslo and its environs. Interviews were conducted in Norwegian, English or French, audio-recorded and transcribed verbatim into English. The analysis was based on a thematic approach, using NVivo software. Interview data were analyzed searching for themes and sub-themes that emerged inductively from the interviews.

**Results:**

Our findings reveal barriers in two main categories when accessing the Norwegian healthcare services. The first category includes difficulties before accessing the healthcare system (information access, preference for doctors with an immigrant background, financial barriers, long waiting time and family and job responsibility). The second category includes difficulties experienced within the system (comprehension/expression and language, the *black elephant* in the room and dissatisfaction with healthcare providers).

**Conclusion:**

Healthcare is not equally accessible to all Norwegian residents. This ultimately leads to avoidance of the healthcare system by those most in need. Lack of seeking healthcare services by immigrants from Sub Saharan Africa may have significant implications for the long-term health of this group of immigrants. Therefore measures to address the issues raised should be prioritized and further examined.

## Background

Sub-Saharan African (SSA) consists of regions economically classified as low-income countries and having some of the worse human development and health indices in the world [1, 2]. Due to the economic situations and political instabilities in some regions of SSA, people tend to migrate to other parts of the world, amounting to 4.15 million sub-Saharan African migrants in Europe in 2017 [3]. The first waves of SSA immigrants migrated to Norway in the 70s, and presently, 916,625 immigrants and Norwegian-born to immigrant parents constitute part of the total population, with 112,786 from 55 countries in SSA. [4]. Oslo has become home for 27.8% of them and has a number of established cultural networks.

Upon arrival, many migrants have better self-reported health compared to the general host, a phenomenon known as “healthy migrant effect” [5, 6]. However, after a period in the host countries the “healthy migrant effect” may wear off, and the health of many immigrants eventually worsen [5]. Recently, immigrant’s disparities in health and access to healthcare services have attracted increased attention in high-income countries [5]. The relationship between cultural and social norms and health care utilization patterns seem to differ between sending and receiving nations [7–9], and there is an ongoing debate whether immigrants benefit equally from services as the non-immigrant [8].

Access to health care services is often one of the indicators of equity in health care provision [10]. Providing health care on equal terms has become a challenge for the health care system all over the world [11–14]. Some individuals do not achieve this fairness because of their social position or other socially determined factors, which in essence negatively affect their health and quality of life in general [15]. Equity in accessing healthcare is a central objective of many health care systems and has been an important buttress of the Norwegian National Health Service. Reducing inequity in health between socioeconomic groups in Norway is the state’s priority, with targets set at local and national levels [16].

The Norwegian health care system is founded on the principles of universal access, decentralization and free choice of provider [17]. It is financed by taxation with minor out-of-pocket payments (co-payments). The Norwegian General Practitioners (GP) are the backbone of primary healthcare (PHC) and gatekeepers for secondary care. All immigrants with a legal residence permit and asylum seekers are entitled to the same health services as Norwegian-born [16].

However, the extent of use of health services among immigrants may vary depending on their health care needs, health care seeking behaviors, the organization of health care in their home country, practical barriers to access in the host country, health literacy, migrant’s status, education level and other socioeconomic factors [15, 18–25]. Examining issues of accessibility to healthcare among immigrants including understanding their experiences in accessing health care, is essential to improving their health.

Immigrants in Norway and other high-income countries have been described to face barriers to accessing healthcare services [5, 26]. Lack of access to healthcare services by immigrants represents a concern for the host countries and the delay in accessing healthcare services may lead to late diagnosis, delayed treatment, and morbidity [27, 28]. Social and economic deprivation has been linked to higher burden and greater risk of disease among some immigrant groups from low-income countries in Norway [4, 29] and other high-income countries [13]. A number of studies among migrants and ethnic minorities have revealed important barriers in healthcare access [30–32]. However, while many quantitative studies explore issues of access among immigrant [33–39], a gap still remains, especially in understanding the immigrants’ experiences to healthcare, as this may be relevant among SSA immigrants.

Existing quantitative studies in Norway have reported findings on the extent of the variation in health care service utilization and the incidence of disease event between various immigrant groups, without giving insights into immigrant’s perceptions [40–42]. Africa immigrants are often examined as a single group, because of their geographic zone, related lifestyles, and health problems. In addition, studies in Norway often grouped African immigrants with immigrants from other regions of the world, in assessing the use of healthcare services [40–45]. Meanwhile, certain factors may have a differential effect on health care utilization between population groups [8, 9], especially as SSA immigrants are confronted with issues of low socio-economic status, language difficulties, coupled with having different cultural beliefs and boundless trust in traditional medicine [46–49]. In addition, this population is different in that, as “blacks”, they often experienced racial discrimination in most walks of life [39, 50, 51] and treated as second class citizens [52].

It is important for the population to get the right services at the right time to ensure and promote better health outcomes. Understanding the accessibility to healthcare among the immigrant population is essential and timely as a guiding phase in improving their health, and knowing their experiences in access to healthcare may be a great step for effective disease management/intervention for better health outcomes. Despite the growing numbers of SSA African immigrants in Norway, little is known about their experiences of accessing healthcare, which is key in generating solutions to enhance healthcare access. This paper, therefore, presents the findings of a qualitative study exploring the experiences of SSA immigrants, to accessing the Norwegian healthcare services.

## Methods

A qualitative research design was chosen to have a detailed understanding of immigrants’ experiences and reflections and individuals’ objectivity with the Norwegian healthcare system.

### Participants, recruitment and data collection

In the recruitment process, we used the snowball technique, where we first identified different informants in a number of established cultural networks including faith-based organizations and cultural groups. The identified informants later recruited their peers. Immigrants from SSA were identified and were informed about the research study and the immigrants and their descendants from SSA countries as described previously [37], with legal residence, at least 18 years of age and willing to participate were included in the study. This study was conducted from October 2017 to July 2018.

The participants were informed of the study objectives through an information letter. They gave both written and verbal consent to participate, and appointments were taken at their conveniences. Because the participants were from many different backgrounds and had different experiences, we included many informants, in order to explore the themes we had and stopped after data saturation.

The primary mode of data collection involved in-depth interviews (IDI) and focus group discussion (FGD), conducted by the first author. These two methods offered the participants the opportunity to share detailed information about their experiences and opinions [53, 54] to healthcare access. We chose these two methods in order to have a better understanding of participant’s experiences both at an individual level and within a group.

The IDIs were conducted in English, Norwegian, or French by the first author. During three of the IDI, a research assistant translated into Somali and Arabic. English and Norwegian were used in the group discussions. Of the 50 participants recruited for the IDI, 47 completed the IDI. The IDIs lasted 45 to 75 min. Two FGD were held with nine participants per group. The FGDs included people from different social background and different SSA countries. The FGDs lasted for 90 to 105 min. The participants held the FGDs and the IDIs at a location of their choice.

The interviews started with questions to gather the general characteristic of the participants. Then, participants were asked to reflect on the barriers to accessing the healthcare system and experiences navigating the healthcare system. The interviews focused on the visits to the general practitioner, emergency room, other healthcare services visited, cognizance of healthcare services, access to health information and the general perception of the healthcare system. After piloting the topic guide for the focus group, with six participants, the research team met to review early transcripts and adjust the topic guide to better capture participants’ perspectives. Because the modifications of the topic guide were minimal, we went back and interview the six participants on the topic we adjusted, so, we included the pilot interviews in the final analysis.

This research study is part of the research project “Access and utilization of healthcare services among immigrants from sub-Saharan African living in Norway”, approved by the Norwegian Regional Committee for Medical and Health Research Ethics (2016/799/REK Vest) and the Norwegian Social Science Data Services (NSD).

### Data analysis

All interviews and FGDs were audio-recorded and transcribed verbatim into English. As an exploratory study, the research did not attempt to test existing theories on barriers to healthcare for immigrants. To ensure trustworthiness, field notes were maintained to document the interviewer’s perceptions and interpretations during each interview. Data were analyzed for themes and patterns, and the themes and sub-themes that emerged from the data [55, 56]. An initial working coding scheme was generated from a consecutive review of the transcripts. Then, with the working coding scheme, we coded a second set of transcripts and revised the theme until no new theme was identified. The codes were later grouped into each theme, and the relationships among the themes were interpreted. The first author conducted the first analysis; and as a method of triangulation, the second author read the transcripts and provided the additional viewpoint of the analysis and the interpretation. NVivo 11 software was used for data management and analysis.

## Results

### Characteristics of participants

Table 1 gives the demographics of the participants for the IDI. Five women and four men participated in the first FGD, while seven women and 2 men were in the second FGD. Participants were mainly migrants from 14 SSA countries, both men and women, aged 18 years and older, with a combination of Muslim and Christian. The majority were between the ages 30–50 years and more than half of the participants, had up to secondary school education. Almost all of the participants were employed and some had attended a professional course and were either assisting in the kindergarten or working in the nursing homes. Some owned private businesses as cosmetic/Afro shops or a restaurant. The rest of the participants were working in a Cleaning company and some unemployed. More than half of the participants migrated to Norway between the late 90s and early 2000 and the main reasons for migration were to seek asylum and family reunification.

**Table 1:** Demographic characteristics of in-depth interview participants

	Total (%)
Total population	47 (100)
Gender	
Women	26 (55.3)
Men	44.7 (21)
Age distribution	
18–30	15.0 (7)
31–40	28.0 (13)
41–50	45.0 (21)
51–60	8.5 (4)
≥ 60	4.1 (2)
Country of origin	
Somalia	23.4 (11)
Sudan	17.0 (8)
Nigeria	10.6 (5)
Cameroon	8.5 (4)
Ethiopia	6.4 (3)
Ghana	6.4 (3)
Senegal	6.4 (3)
Eritrea	4.3 (2)
Uganda	4.3 (2)
Gambia	4.3 (2)
Liberia	2.1 (1)
Congo	2.1 (1)
Burundi	2.1 (1)
Tanzania	2.1 (1)
Social status	
Married	78.7 (37)
Single	17.0 (8)
Divorce	2.1 (1)
Undisclosed	2.1 (1)
Education	
No formal education	12.8 (6)
Primary	6.4 (3)
Secondary	36.2 (17)
High school	29.8 (14)
University	23.4 (11)
Employment status	
Employed	74.5 (35)
Unemployed	19.1 (9)
Retired	4.3 (2)
Undisclosed	2.1 (1)
Years living in Norway	
Born in Norway	2.1 (1)
≤ 10 years	36.2 (179
11–20 years	46.8 (22)
≥ 20 years	17.0 (8)

**Table 2:** Presentation of themes and sub-themes

Themes	Sub-themes
Difficulties before accessing the healthcare system	• Information accessibility • Preference for doctors with an immigrant background • Financial barrier • Long waiting time • Family and Job responsibility
Difficulties when in the healthcare system	• Comprehension/expression and language • The “Black Elephant in the room” • Dissatisfaction with healthcare providers

The reasons for seeking health care services and experiences for different healthcare services visited varied among the participants. Participants faced barriers to system access, healthcare providers and navigations related to their needs for multiple services. The barriers were in two categories: difficulties prior to accessing the healthcare system, and difficulties experienced once in contact with the system (Table 2). The findings reveal categories of accessibility barriers of concern to the participants. Contextual and societal factors hindered their efforts to seeking healthcare. Here, we present the specific concerns raised by participants as they relate to each of the categories. Verbatim quotes have been selected from the IDIs and FGDs to apprehend the perceptions, and experiences shared by the participants with respect to access to the Norwegian healthcare system.

### Difficulties before accessing the healthcare system

#### Information accessibility

The participants were aware of the existence of the GP, emergency room and the referral scheme, but were unaware of preventive and mental health services and counseling. In addition to the lack of knowledge about the availability of the existing healthcare services, those with psychological problems for example substance abuse, trauma (missing their children collected by the child protective services) did not know where to seek help and they were unaware that they could be referred to see specialist for their psychological problems. Participants did not know the right kind of services that should be used when different health needs arose. Had the participants had this information’s it would have guided them for appropriate health decisions.

*“I don’t know if they are available and where to get them. Sometimes I go about reading through the net and asking people if they know if a particular health service does exist in Oslo. I am really lost in terms of knowing and navigating the Norwegian healthcare system.” (FGD, group 2)*

*“I have not been to the hospital for depression because I do not know if the service is here in Oslo and even if it is here where can I find it. It is very difficult to know where mental health services are in Oslo. I don’t know where to start finding the hospital [ … ]”. (Interview, participant C7)*

Health information available only in Norwegian at certain health facilities further intensified the problem. Some participants complained that they lack health-related information in their language and perhaps in English. Participants expressed they sought information from relatives, Google and other online sites for answers to their health needs.

*“Everyone struggle to get information from the left or right, either by asking friends who are health professionals or read from Google.” (Interview, participant D10)*

Regarding the accessible information, participants emphasized that the government pay much attention to cancer prevention, and less to sicknesses believed to be common among the African immigrants in the community, including vitamin D and iron deficiency, hypertension and diabetes. They requested the government should inform them of the preventive measures of the ailments said to be common in the community.

*“To get information about health is difficult. I only get information about cancer but not for HIV, high blood and diabetes. They are also very dangerous diseases. The government should also send us a letter about them. I also need information about vitamin D and iron. Many of my friends are suffering from a lack of vitamin D and iron. The government should tell us why many black people are facing that problem […]” (Interview, participant A8)*

*“My father finds it difficult getting health information on diabetes. We only hear from friends or people who have lived here for a long time.” (FGD, group 1)*

Many participants felt that the physicians place more values on medications than advice on disease prevention.

### Preference for doctors with an immigrant background

Many participants felt that the immigrant doctors trained in or out of Norway would be an ideal solution to their accessibility problems. Although most immigrants will prefer doctors with an immigrant background, their top priority was immigrant doctors from Africa. Their preference stemmed from the respect, attention, and treatment they perceived to get. Their disappointment and frustration with doctors with non-immigrant background urge them to seek alternatives to the official public system.

*“I get respect from my doctor [the immigrant doctor] and he is very friendly to my family. I am very satisfied with him. He cares for me and refers me to a specialist. He does a lot of investigation on me [ … ]. He visits us at home and we can call him whenever we want and can go at any time to see him [ … ]. He has never refused to see us on the same day. He will look [physical examination] at us and take his time to know about the start of the sickness.” (Interview, participant A5)*

*“If my children are sick I don’t go to a Norwegian doctor [non-immigrant background], I just call my Sudanese friend who is a doctor here and things will go very fast.” (FGD, group 1)*

*“I prefer going to see a private doctor, especially those from Nigeria.” (Interview, participant E1)*

*“I really will prefer a foreign doctor especially one from Africa.” (Interview, participant B6)*

Although few participants had Norwegian doctors, they were very convinced that a physician with the same ethnic background would understand them better than the Norwegian doctors would. Majority of the participants changed their family doctors to an immigrant doctor and it was discussed hotly that the government should employ doctors and specialist from their region of origin.

### Financial barrier

Economic affordability is in relation to the direct cost of receiving healthcare services. The participants lacked the understanding of their entitlement to free healthcare. Being entitled to free healthcare is confusing to many, and the issue of co-payment for doctor’s visit is a source of distress. Although healthcare is subsidized, almost all the participants wanted free healthcare. Some participants prayed not to get sick because they could not afford the co-payment. They expressed the patient’s co-payment for a doctor’s visit be let off. Dental care and physiotherapy were perceived to be expensive and unaffordable.

*“The dental care services here are unaffordable. One needs to look for alternative treatment when needing dental care. Some Africans take the bus and go to the Czech Republic for dental care.” (FGD, group 2)*

*“Physiotherapy is expensive for me. Some days I do not go, I skip until I can afford it. It cost me a lot just for single physiotherapy treatment.” (Interview, participant E7)*

Our findings revealed that because of the cost of healthcare, immigrants may either forgo treatment, may travel abroad to nearby countries for cheaper treatment, may seek alternative traditional treatments or may wait to seek care during their next trip to Africa.

*“ … The healthcare in Turkey is cheaper than that of Norway. So, if my illness is serious I go to Turkey and if it is not that serious I consult with a private doctor … the money will pay for healthcare in Turkey is almost the same as the money we pay in Norway for consultations and laboratory fees.” (Interview, participant A4)*

*“The treatment for dental care is very expensive. … I cannot visit the dentist because it is expensive. I use some herbs on the hole in my teeth to relieve the pains. I am waiting for my next trip to Ghana so that I can visit a dentist for treatment and filling.” (Interview, participant A7)*

### Long waiting time

Long waiting time was a repeated theme in all the interviews. Many participants expressed dissatisfaction with the long waiting times for doctors’ appointments, referral, at the emergency room and some public services, due to bureaucracy procedures.

*“They take a long time in diagnosis. The results of my test took a long time. I was without treatment until when I got the result of the test from the laboratory [ … ]. It can take about 5 to 6 months to see a specialist in this country.” (FGD, group 2)*

*“The emergency room may have many patients to attend to and waiting for treatment can be a big problem.” (Interview, participant D8)*

Participants discussed that certain conditions would have been avoided if they sought care earlier. They expressed that long waiting time prolongs the process of obtaining treatment and sometimes can be life-threatening. They believed that referral to a specialist was too long for a wait and some boycotted to the private sector or traveled abroad for treatment. Migrants were aware of the delay in treatment, caused by bureaucratic procedures of the public health system and this caused frustration and feelings of discrimination and exclusion.

*“Here [Norway] waiting for a long time to get an appointment can lead to the death of a patient.” (Interview, participant B7)*

*“It took a long time for my wife to get ultrasound [ … ]. I was so afraid of complication. People going in for an ultrasound, MRI, and x-ray always complained of having a long time to wait before the procedures.” (Interview, participant A8)*

*“The nurses in the front desk told me that my doctor could not see me until after 2 weeks because she has many patients [ … ] I was spitting out blood.” (Interview, participant C1)*

### Family and job responsibility

Culturally, most African believes in “holding each other’s back” or assisting/being there for each other. Family responsibility and job security were prioritized to healthcare seeking.

*“Family responsibility as household chores and sometimes our jobs are a barrier to access to healthcare. You know as Africans, we still maintain our responsibility in caring for the family. Our family comes first before ourselves … our family comes first.” (Interview, participant D6)*

*“I am a single mother and I have to only attend hospital appointment when my children are at school. When they are at home, especially during the holiday I cannot attend an appointment because there is no one to take care of them.” (FGD, group 2)*

According to most of the participants, financial constraints mean limitation to an entire family. Missing a job means starving or somewhat not taking care of several family members in their countries. It was expressed that the employer did not hinder care, but may indirectly have an impact.

*“I fear to lose my job because my boss is also complaining that I am taking leave all the time. I am afraid that one day he may say I should stop working and how will I take care of my family back in Africa.” (Interview, participant A10)*

*“ Our job is our life and the life of those back at home, so we cannot leave our job for simple hospital treatment for body pains.” (FGD, group 2)*

For the reason that some participants were into cleaning and newspaper/packages delivery services, some could barely find the time to meet up with GP appointments, because of work intensity or exhaustion from their jobs. But, they ensured their children did not miss any hospital appointments.

*“Sometimes I barely find time for the appointment, because of the nature of my job. I start very early to distribute or take parcels to various destinations. [ …] Either I forget to go to the appointment or I cannot find time to attend because of work.” (Interview, participant D2)*

### Difficulties when in the healthcare system

#### Comprehension/expression and language

Communication or expression difficulties represent a significant barrier to receiving appropriate healthcare. It was a recurrent theme in all the interviews and affected all aspects of the healthcare, from accessing, understanding health-related information to receive the right diagnosis and treatment. Participants expressed apprehension regarding their ability to convey their health concerns in Norwegian, the physician’s inability to comprehend their health concerns as well as their capability to interpret medical directions provided by the physicians. Some participants expressed that because of the communication barrier or because of the lack of comprehension for both the patients and the doctors, they spend a lot of time trying to get their symptoms through to the health professional. Although the State takes financial responsibility for interpreters in the health sectors, the majority of the participants are unaware and do not take advantage of this offer, but rather rely on their basic knowledge of Norwegian.

*“With a non-immigrant doctor, he will ask you the same question more than two times. I do not know if they do not understand the Norwegian I speak, or they do not understand the symptom. I cannot tell if it is my accent or if they do not understand what I am saying. Sometimes I feel very embarrassed. They ask and ask and ask. Maybe our accent is difficult to understand. I speak in Norwegian, yet the doctor will ask that I repeat myself.” (FGD, group 1)*

*“It takes a long time for them to understand me. I do not speak good Norwegian. The doctor understand English but I do not understand English and when I speak little Norwegian they too do not understand. I have to repeat myself over and over […] ” (Interview, participant D5)*

In addition of taking time in expressing themselves in the best language possible, some participants could not discuss in detail their symptom to the doctors, because the doctors were always in a rush to attend to the next patient. They bothered that health information’s at the health services and the chemists are inscribe only in Norwegian.

*“Every information’s in the hospital or on medication packages are written in Norwegian and it makes things very difficult for us. I know that educated people do not suffer, but for those who can barely speak nor understand the language is a very big challenge.” (FGD, group 1)*

*“Medications instructions are labeled in Norwegian and how can we follow the instructions written on the leaflets.” (Interview, participant A9)*

### The “Black Elephant in the room”

Many participants felt discouraged because they perceived the care providers did not seem interested in them. They felt ignored and being treated as second-class citizens. They believed the care providers paid less attention to them than they did to other patients of a different race

*“They pretend not to understand you [someone] and they ignore your presence and concentrate on different patients that are white.” (Interview, participant A4)*

Some participants also felt the care providers were scrutinizing them. Those that experienced this said they were so certain not to consult with the same doctors in the future.

*“[…] she will ask me many questions concerning my private life, like what brought me to this country and why don’t I go back to Uganda and find a better job.” (Interview, participant D9)*

*“Sometimes when I go to my doctor, he immediately asks the questions: what are you sick of? What do you want? Did you come for sick leave? The first thing when they see me, they think I am there for sick leave.” (FGD, group 1)*

Most of the participants were equally worried about why HIV test was often among the list of laboratory test check. According to the participants, it was obvious they were being suspected of having infectious diseases, which were affirmed by the facial expression and actions of the healthcare providers.

*“I was surprised that my doctor told me it is good that I do not have HIV and that I should take care of contacting it and if I want to do further test, I can do that after 6 months. What is the problem with these guys [doctors]!. Is it HIV test I went for!. I was so angry that I wanted to explode but for a fact, I respected myself. Can they do that to a Norwegian?” (Interview, Participant D3)*

*“One day I had anemia and when I went to the hospital, the doctor listed a very long list of test for me to do, including HIV.” (Interview, participant C4)*

In a particular case, a nurse was said to double her gloves before collecting the participants’ blood. Another participant reported that the nurse gazed into her eyes before wearing her gloves. It was all perceived as not wanting to have direct skin contact with them or avoiding contamination.

*“You need to see them [nurses] when they want to physically examine an African child. Some do double their gloves and the expression on their faces shows they are avoiding to have direct contact with the child.” (Interview, participant D3)*

*“One day I was in the hospital and I was the next to give my blood for a blood test, the nurse that was collecting the blood was not wearing gloves. I saw it because I was right at the door and she asked me to give her some minute to finish with the patient in the room. When she finished collecting the blood and it was my turn, she gazes at me straight in the eyes and immediately pick up her gloves and wore them before collecting my blood.” (FGD, group 2)*

It is worth emphasizing that the recurrent inclusion of HIV test among other test and the use of double gloves were highly perceived as suspecting them of being contagious. Most believed that they were being discriminated because they were black and from Africa. Participants perceived this to be disrespectful, unfriendly and an idea that has been preconceived of Africans. They felt neglected and isolated and supposed the healthcare providers preferred talking to patients of different ethnic background and race.

*“The nurse that was supposed to perform the dialysis was ignoring me during the process and was paying attention to the Norwegian guy that was on the other bed. We were two but she was only asking the other guy how he was feeling while ignoring me.” (Interview, participant D7)*

*“The doctors treat the African differently. We are not always greeted in a friendly manner as compared […] the people are always biased towards Africans. They treat us different and they talk to other white people with respect, but with us, they are very rigid. They do not smile and only send us to do the test or prescribed medications. No physical examination.” (Interview, participant D2)*

### Dissatisfaction with care providers

Expectation regarding treatment was hotly perceived and discussed. Many participants described their frustration because of incorrect diagnoses and inappropriate treatments. One participant described being misdiagnosed of cancer, and she believed this was because of improper diagnostic procedures.

*“I had swollen throat last year and went to see the doctor and he asked for series of test to be carried out and finally after some time, he said I have no problem and I was given pain medication. The problem and the pains continued and I went back to him he called some other doctors in and they examined my neck and they were murmuring and after some minutes they said I have to see a specialist for he has to examine me for cancerous cells. It took another 3 weeks and they said I still have to go for further test. My husband said I should travel to Germany to see a Nigeria doctor and I did, it took just a single examination and the result came out to be a problem of the thyroid.” (Interview, participant B6)*

The participants’ skepticism of the care provider’s skills and subsequently the treatment they received emanate from the repeatability of treatment regimens, hospital revisits and lack of sufficient time to express their symptoms to doctors’ understanding.

*“When I had a kidney problem, the doctor did not give me the right medication. They tested me with a lot of medication and the case was getting worse. It actually took them about 8 months before they started the real treatment.” (FGD, group1)*

*“The baby was sick and I took him to see the doctor […]. The doctor could not diagnose what was wrong with the baby. The baby’s health was not improving so I had to visit and revisit the hospital for the third time.” (Interview, participant B6)*

Many participants expressed that in contrast to their home country, physicians in Norway do not do a physical examination, but rather rely on a description of the patient’s symptoms in making an initial diagnosis.

*“The doctor in the “legevakt” [emergency room] never touched me [physical examination]. They only allow you to speak but they can never touch you. I was asked to go and rest and take water and eat well. When I got back home the problem continues and it became chronic […].” (Interview, participants A5)*

Participants expect the routine examination of vital signs as they experienced in their countries, where, patient’s pulse and temperature are checked during every consultation. They expressed that the doctors in their home countries hold patient’s hand, check the blood pressure, temperature and check the eyelids for signs of blood deficiency. They were distressed that the doctors in Norway focus more on their computers to write down what the patients tell them. Their expectation is for the physician to make a diagnosis based on physical examination. Participants felt that Norwegian doctors spend less time with them. Short and hasty consultation with the physician-led to disappointment, lack of confidence in the physician and distrust in the healthcare system.

Participants were worried that the doctor does not trust their words. The myths of drug trials as habitual in Africa prompted some participants to be skeptical of the prescribed drugs, to be for “drug testing” or “experimental drugs”.

*“I do not trust the doctors here. I am always afraid that they are testing drugs on us and may use us for experiments. I am very careful when taking the medication. I also call someone to read all the things written on the medications to confirm before taking the medication.” (FGD, group 1)*

The participants expressed that they are not trusted enough to be given sufficient sick leave to address their health issues. They voiced that no matter the severity of their ill health, they wouldn’t be given more than 5 days of sick leave. This caused tension between the doctors and the patients and eventually boycott. They felt as if the physicians were more concerned with the financial loss of the Norwegian Labour and Welfare Administration (NAV) scheme than their health needs.

## Discussion

Providing health care on equal terms has become a challenge for the health care systems around the world [5, 11, 12, 14, 57, 58]. This study highlights that SSA immigrant’s in Norway face challenges in accessing healthcare services both prior to accessing the healthcare services and when in contact with the healthcare system. Our data demonstrate that utilization of healthcare is not only influenced by affordability but also by lack of information about the existence and adequate use of healthcare services when the need arises. Similar to other studies, non-proficiency in the host country language and low health literacy impede immigrants from accessing healthcare [59, 60] Our study highlighted the need for easily understandable information in English and other relevant languages, on the Norwegian healthcare system to be made available, specifically about disease and preventive measures for appropriate health decisions.

In addition, the direct cost of healthcare was cited as a barrier to healthcare services and reimbursement bureaucratic procedures was said to be complicated. Even though the Norwegian government ensured health insurance coverage and financed the healthcare system, the out of pocket expense is considered to be high, for those with economic hardship and this affects access to primary healthcare. Researches have shown how lack of sufficient finances significantly affects access to healthcare, both for the immigrants and the non-immigrants [61]. Demographic characteristics, most especially low income have also been shown to play an important role in persisting disparities in access, despite the presence of health insurance coverage [62]. Our findings revealed that lack of sufficient finances or income might force SSA immigrants to seek alternative or self-treatment, and may mean that most SSA immigrants in Norway might not be able to make payments for high-cost procedures such as eye and dental care and physiotherapy.

Furthermore, health professional support to the SSA immigrants is of great importance for a positive encounter with healthcare. Immigrants’ preference for immigrant doctors is partly a consequence of participants perceiving the Norwegian health professional show less respect and interest in them. Similar to our study, other researches highlighted that immigrants have been distraught because health professional show no interest in them [63], spend less time with them during the consultation [64] and professionals are often in a rush to attend to other patients [65]. It is of importance for the health professional to support SSA immigrants, especially when in contact with the health care system, for reason that they are from countries with complex health issues and most of them might have migrated from countries with political instability and cultural practices, which might have subjected them to physical and mental health-related issues.

Other factors were also reported in this study regarding difficulties attending appointments, including long waiting time and family and job responsibility. Long waiting time is not unique to the immigrant [66, 67]. Long waiting time has led to patients’ dissatisfaction with health care and death of patients [67, 68]. If countries with limited means can achieve the virtual absence of waiting lists, then what excuse can there be for countries such as Ireland, the UK, Sweden or Norway to keep having waiting list problems?. However, reducing patients wait time may contribute to better health outcome [69] and should be a priority for the Norwegian health care. Family and job responsibility were emphasized to influence immigrants access to healthcare particularly among married immigrants and those with children, who perceived conflict between their own care and to the care for their family and protecting their jobs.

In addition to challenges faced before accessing the healthcare system, immigrants experiences while in the healthcare system were also enumerated. Similar to other research [30, 32], communication between the health professionals and immigrants is important, and that insufficient language knowledge acts as a barrier. Communication difficulties affected SSA immigrants’ ability to interact with the healthcare system once in contact with the system. The use of interpreters during consultation would be of an advantage in reducing communication barriers. Immigrants have significant difficulties with health literacy and can accordingly be challenge by intercultural communication barriers when accessing healthcare and making sense of the related health information. This could lead to misguidance and subsequently health errors and health problems. Health literacy has clearly shown to have an impact on health decisions [70]. These difficulties compromised the quality of care on a number of levels such as discouraging SSA immigrants from accessing care, making it difficult to describe symptoms and disease prevention. Language difficulties among non-western ethnic minorities in Norway accounted for dissatisfaction with the physician and lower attendance in health surveys [71]. The efficacy of care, which included interpersonal communication and clinical effectiveness, could be linked to an extent to language problems. It is important that the immigrants should have proper and effective communication with the care providers and English as a second language instruction have been shown to improve health outcome [72].

Perceptions of discrimination and negative stereotypes from health providers emerged as a barrier to access. This reflects similar findings, where, other research has shown that black patients and low socioeconomic status influences physicians’ perceptions and attitudes towards patients, and that physician’s view patients from ethnic minorities and of low-economic status more negatively than the white patients and patients with high economic status [73, 74]. The participants felt the health professionals were judgmental because they were black. Asking participants to do HIV test, the use of double gloves and gazing straight into the participant’s eyes before health professionals wore their gloves could be a sign that the care professionals were afraid of contamination. The speculation could be that, since the immigrants migrated from areas of high endemic of infectious diseases, they might be carriers. But the question is, why did those born in Norway also experience this? Participants’ perception of being a black and the paradox of relating “African with infectious diseases” could be the reason of health professional attitudes towards them, or it could be the idiomatic expression “Black elephant in the room” which has caused a lot of frustration and controversy among the black community and this has often been overlooked in codified social interactions [75]. Health professional putting an extra pair of gloves during a hospital visit [58] has been shown. Discrimination and mistreatment because of skin colour, race, ethnicity, name, country of birth, language or religion background have been propounded as drivers of racial/ethnic inequities in healthcare [76, 77]. The negative impacts of racism on physical and psychological health are well verified, mostly focusing on measures of individual personal experiences of racism [77–79]. Racial discrimination experienced within the healthcare setting may affect how people perceive the healthcare system, how they engage with health services and care providers, as well as the patterns and quality of their healthcare access [80, 81]. Experiences of racism may potentially influence patient satisfaction, levels of trust, and perceived quality of healthcare interactions, and consequently may influence their future patterns of health service use [81, 82].

Our participants perceived health professionals with immigrant backgrounds and private healthcare providers are more willing to listen, talk and explain things to them. They expressed that discrimination and miscommunication were more frequent in the public sectors. This may be related to the fact that physicians’ practices are known to be influenced by healthcare remuneration scheme with doctors spending less time with fee-for-services patients [83, 84]. Although our research does not directly reflect on delivery of quality of care, our findings suggest that negative stereotypes and lack of cultural awareness may inadvertently lead to inequalities in the quality of care among SSA immigrants, compared to the rest of the population. Culturally appropriate healthcare development could possibly address these factors [85].

Participant’s expressed dissatisfaction with the healthcare providers. The perceived unskillfulness of the health professional may possibly stem from the cultural differences with that of the host country, and this might have impelled the immigrants to prefer consulting with immigrant doctors. A previously reported [85], culturally appropriate care may be crucial to address cultural differences concerning diagnosis, symptomology and the understanding of the health investigation system in Norway. Although cultural appropriateness alone may not be sufficient for reducing healthcare disproportions, it nonetheless remains one of the most significant implements in addressing health disparities in society [86]. Finally, distrust of the healthcare providers and the healthcare system, in general, raised a lot of skepticism, especially as participants were worried about the issue of “drugs testing”. The issue of “drug testing” may have been conceived prior to migration. However, health care providers can build trust by being transparent about the decision underlying the treatment and perhaps being more explicit in explaining why a physical examination may not be required could be helpful. A similar study in the Netherlands among Ghanaians revealed that participants perceived the drugs prescribed to them were for “test prescriptions” [31]. Similar to other studies, participants equally felt that some health workers were more concerned with the financial benefits of the state, rather than an issue concerning their health [87].

From a theoretical perspective, the finding of this study adds records to the capability perspective in healthcare research. Although the literature on healthcare research is broad, it does not take into consideration Africans immigrants from the sub of the Sahara. The findings of this study bridge the knowledge gap of SSA immigrant’s access and use of the Norwegian healthcare system. It provides a better understanding of how SSA immigrants access and use healthcare services in Norway. As asserted by some analysts, only when the health needs of immigrants are addressed, would the host countries be able to support the health of the country as a whole [88]. The study findings may contribute to generating solutions for a positive encounter with the healthcare services for better health outcome and general wellbeing of SSA immigrants.

## Conclusions and recommendations

This study makes it clear that while the Norwegian healthcare system is founded on the belief of universal coverage, healthcare is not necessarily equally accessible to all Norwegian residents. Despite having a well-funded system marginalized SSA immigrant group are faced with multiple barriers before they reach out for care and when they are in the healthcare system. This ultimately results in avoidance of the healthcare system by those most in need and that may have significant implications for the long-term health of SSA immigrants in Norway.

This represents an important area of future investigation of the issues raised and factors identified in our study. Future research must further the understanding of these factors that hinder health, should guide policy development and identify areas for improvement. More qualitative research should attempt to explore how migrants understand their health and health norms including the time they should access healthcare and what type of healthcare. Future research should look at bringing together and advancing both epidemiological and qualitative findings.

As the rate of immigrants in Norway and high-income countries are on a rise [4], and as the structure of the immigrant population become increasingly diversify, more immigrant may find that their access to medically necessary services is compromised. This will indicates the need for a healthcare system and healthcare policies that are more sensitive and responsive to the increasing diversity of the Norwegian population. It will require health policy to move beyond multicultural rhetoric.

## Data Availability

The datasets generated and/or analyzed during the current study are not publicly available due to confidential reasons. The datasets are under the management of the University of Oslo, but are available from the corresponding author on reasonable request.

## Abbreviations

FGD: Focus group discussion
GP: General practitioner
IDI: In-depth interview
NAV: Norwegian Labour and Welfare Administration
NSD: Norwegian Social Science Data Services
SSA: Sub-Sahara Africa.
